# A Controlled Study on the Correlation between Tear Film Volume and Tear Film Stability in Diabetic Patients

**DOI:** 10.1155/2016/5465272

**Published:** 2016-02-29

**Authors:** Iman M. Eissa, Noha M. Khalil, Heba A. El-Gendy

**Affiliations:** Department of Ophthalmology, Cairo University, Cairo 11562, Egypt

## Abstract

*Purpose*. To assess the tear film quantity and correlate it with the quality and stability of the tear film in diabetics and compare them to age matched controls.* Introduction*. Diabetes affects tear film parameters in multiple ways. Poor metabolic control and neuropathy are postulated factors. To further understand how diabetes affects tear film parameters this study was conducted.* Subjects and Methods*. Tear meniscus height was measured by anterior segment OCT, along with tear thinning time, a subtype of noninvasive tear break-up time, and blinking rate per minute which were all recorded for 22 diabetic patients. Correlations between these tear film parameters were studied and then compared to 16 age matched controls.* Results*. A statistically significant difference was found in blinking rate between the diabetic and the control group (*P* = 0.002), with higher blinking rate among diabetics. All tear film parameters were negatively correlated with duration of diabetes. A positive correlation was found between tear film volume and stability.* Conclusion*. Diabetes affects the tear film in various ways. Diabetics should be examined for dry eye signs even in absence of symptoms which may be masked by associated neuropathy. Duration of diabetes has an impact on tear film status.

## 1. Introduction

Tear film studies have progressed a lot in the past few years, from the traditional quantitative tests like Schirmer test, and tear meniscus height measurement, and the qualitative test of tear break-up time (TBUT), to anterior segment optical coherence tomography (AS OCT) measured tear film height and the dynamic tear film studies frequently used nowadays using computerized videokeratoscopy [1].

A lot of studies pointed to the fact that diabetes mellitus greatly affects the tear film function and stability. Decreased Schirmer 1 test values and shorter BUT were positively correlated with the subjective severity of dry eye symptoms in type 2 diabetic patients. Moreover, the decreased tear film function was found to be more severe in patients with PDR than in those with NPDR [2].

Other studies tested the corneal sensitivity, corneal epithelial integrity, and conjunctival epithelium (using impression cytology) and showed that the degree of keratoepitheliopathy was marked, and the corneal sensitivity, TBUT, and tear secretion were all significantly reduced in the diabetic patients. Conjunctival impression cytology showed conjunctival squamous metaplasia and lower goblet cell count in diabetic patients. All these parameters were related to the poor metabolic control, the presence of diabetic neuropathy, and the stage of diabetic retinopathy [3].

In fact, a recent study stated that tear film instability could be a marker of, or rather a predictor for, the occurrence of diabetic neuropathy in type 1 diabetes patients. Misra and coresearchers found a positive correlation between tear film stability and corneal subbasal nerve density (measured by corneal in vivo confocal microscopy) which was statistically significant (*P* = 0.04); they also found that decreased tear film stability was associated with increasing age and duration of diabetes [4].

Recent tests for assessment of the tear film quantity include AS OCT measurement of the tear wedge area, or the tear meniscus height of the inferior tear meniscus. Other tests for the assessment of tear film stability include the noninvasive BUT and its subtype, the tear thinning time (TTT), which measures the time taken for the tear film to get thinned out even before the actual break-up of the tear film occurs. The TTT has the advantage of not having to instill any eye drops or fluorescein dye prior to the test, which in itself can cause reflex lacrimation in some people and thus affect the accuracy of the test [5].

Another indicator for tear film function is the patient blinking rate per minute which was found to correlate positively to the severity of subjective dry eye symptoms. Dry eye subjects were found to have significantly higher blinking rates. Reduced and incomplete blinking along with increased tear film break-up during normal visual tasks may explain the increased level of ocular discomfort symptoms reported at the end of the day, particularly in dry eye patients. The normal average resting blinking rate per minute is 17, increasing to around 26/min during conversation and decreasing to around 4.5/min while reading [6, 7].

Tear film function and stability in diabetic patients are an area of ongoing active research. To our knowledge, a few studies have investigated the correlation between the quantity and quality of tears [8, 9] but a few or almost no studies examined this correlation in diabetic patients and compared them to age matched controls.

The purpose of this study is to study the tear film quantity (represented by AS OCT measured inferior tear meniscus height) and correlate it with the quality (represented by the TTT for stability, and patient blinking rate per minute) in diabetic patients and age matched controls.

## 2. Subjects and Methods

The study was done in accordance with the ethical standards in the Declaration of Helsinki 1964 [10]. An informed consent was taken from all patients before participating in the study.

In this case control prospective series, 22 diabetic patients (9 males and 13 females), that is, Group A, and 16 age matched controls (7 males and 9 females), that is, Group B, were recruited from Kasr Al Aini (Cairo University Hospital) Ophthalmology and Diabetology out-patient clinics.

The age of the patients ranged from 40 to 70 years (mean 57.22 ± 8.5 SD) for the diabetic patients (Group A) and from 30 to 77 years (mean 57.12 ± 12.22 SD) for the nondiabetic group (Group B).

A full history was taken, including detailed ocular and medical history, as well as a thorough full ophthalmological examination. A routine laboratory work-up was also done for all patients.

The inclusion criteria for patients were as follows:

- A history of diabetes mellitus for a duration ≥5 years for cases in Group A and no history of any systemic disease for group B.
- No history of chronic eye diseases and/or previous ocular surgery.
- No symptoms of dry eye.
- No history of associated systemic conditions.
- No use of systemic or topical medications that may affect the tear film secretion like parasympathomimetics or parasympatholytics.

The patient blinking rate per minute was measured in all patients, by the same observer who attended during all patient examinations and was asked to count the rate of blinking while the patient was having a routine conversation with his doctor.

For assessment of tear film quantity the tear meniscus height (TMH) was measured by the spectral domain optical coherence tomography (RTVue100-2, Optovue, CA, USA) with the cornea-anterior segment lens long attached. Each patient was placed at the chin rest of the AS OCT machine. The tear meniscus height (TMH) was measured through applying a vertical line scan passing the cornea, the inferior tear meniscus, and the lower eyelid at the 6 o'clock position. In the resulting triangular image of the inferior tear meniscus, the height of the limb opposite the acute angle formed between the inferior cornea and the lower lid margin was measured manually using the machine calipers and expressed in microns.

For the assessment of tear film stability, a subtype of the noninvasive BUT, the tear thinning time (TTT), was performed for all cases. Each subject was instructed to place their chin on the chin rest of the keratometer (CIOM SRL, Milano, Italy). The patient was then asked to blink several times and then open his eyes and stop blinking. The observer then counted the time elapsed in seconds between the last blink and the earliest change in shape of the keratometer's mires reflected upon the patient's corneal surface. The time was then recorded in seconds. The tear thinning time is usually normally shorter than the TBUT, as tear film thinning happens seconds before actual tear break-up.

For each measured parameter, three successive readings were taken for each patient, and the average was taken for each case.

Data were statistically described in terms of mean ± standard deviation (±SD), median and range, or frequencies (number of cases) and percentages when appropriate. Comparison of numerical variables between the study groups was done using Mann-Whitney *U* test for independent samples. For comparing gender, Chi square (*χ* ^2^) test was performed. Correlation between various variables was done using Pearson moment correlation equation for linear relation in normally distributed variables and Spearman rank correlation equation for nonnormal variables/nonlinear monotonic relation. A *P* value less than 0.05 was considered statistically significant. All statistical calculations were done using the computer program SPSS (Statistical Package for the Social Science; SPSS Inc., Chicago, IL, USA).

## 3. Results

This is a nonrandomized controlled case study, where twenty-two diabetic patients (Group A), attending the out-patient clinic for routine checkup, who met the inclusion criteria for the current study were recruited.

The obtained data under study were compared to those obtained from a control group of nondiabetic patients (Group B), who still met the inclusion criteria for the current work.

The demographic data of patients enrolled in the study is summarized in Table 1.

The duration of diabetes in group A patients ranged from 5 to 30 years duration with a mean duration of 13.5 ± 7.5 (SD).

### 3.1. The Tear Thinning Time (TTT)

TTT was found to range from 9 to 22 seconds with a mean value of 14.36 sec ± 3.38 in the diabetic patients (Group A), as compared to a mean value of 14.75 sec ± 3.43 in the control group (Group B); the difference between the two groups was found to be statistically insignificant (*P* = 0.6).

Moreover, the recorded mean value was 13.85 sec ± 2.99 in the diabetic females in Group A, as compared with a mean value of 15.11 sec ± 3.95 in the diabetic males within the same group; the difference was noted to be of no statistical significance (*P* = 0.5).

### 3.2. The Blinking Rate

The rate of blinking per minute was recorded to vary from 18 to 40 blinks/min with a mean value of 25.32/min (±6.15 SD) in Group A patients as compared to a range from 15 to 24 blinks/min with a mean of 20.44/min (±2.25 SD) in group B; the difference between the two groups was found to be statistically highly significant (*P* = 0.002).

Moreover, the rate of blinking was found to be lower among diabetic females when compared to diabetic males in Group A, with mean rates of 22.85/min (±3.75) and 28.89/min (±7.35), respectively. This difference was statistically significant (*P* = 0.02).

### 3.3. The Tear Meniscus Height at the Centre (TMH)

The TMH ranged from 163 to 740 *µ*m with a mean of (365.64 ± 148.6 SD) in Group A patients as compared to a range from 171 to 727 *µ*m (356.38 ± 155.75 SD) in Group B patients, with no significant statistical difference (*P* = 0.96).

Regarding the sex differences, the TMH was found to be less in diabetic males as compared to diabetic females in Group A with a mean value of 349.56 *µ*m ± 140.07 and 376.77 *µ*m ± 158.89, respectively, although the difference was statistically insignificant (*P* = 0.81).

### 3.4. The Correlation between the Studied Variables in Group A

Generally, a negative correlation of varying strength was found between the age of the patient and the three studied variables (the TMH, TTT, and blinking rate/minute) as shown in (Table 2).

A strong negative correlation (*r* = −0.625) was found between the age of patient and the TMH in microns, a correlation that was found to be statistically highly significant (*P* = 0.002).

The weak negative correlation between the age and the rate of blinking (*r* = −0.39) was of no statistical significance (*P* = 0.06); however it was close to being significant. The weak negative correlation between age and TTT was of no statistical significance (*P* = 0.67).

Upon correlating the three tear film parameters with each other, a weak positive correlation (*r* = 0.23) was recorded between the TTT and blinking rate/min (representing stability) and the TMH in microns (representing quantity), although it was statistically not significant (*P* = 0.28).

The rate of blinking was found to be positively correlated to the TTT, again with no statistical significance (*P* = 0.83).

A negative correlation was elicited between the studied tear film parameters and the duration of diabetes. This correlation was of statistical significance regarding the blinking rate and TMH (*P* = 0.03), although it failed to achieve a statistical significance regarding TTT (*P* = 0.35).

A summary of the correlation between the different studied parameters in Group A is elaborated in Table 2.

### 3.5. The Correlation between the Studied Variables in Group B

A weak negative correlation between the age of the patient and TTT as well as TMH was elicited (Table 3) that was noted to be statistically nonsignificant. However, a weak positive correlation between the rate of blinking and age was noted as well, again with no statistical value.

Moreover, a weak negative correlation was recorded between TTT and blinking rate/min (*r* = −0.21) that was considered to be of no statistical significance (*P* = 0.4) as compared to the moderate positive correlation between TTT and TMH (*r* = 0.46) that was noted to be statistically close to being significant (*P* = 0.069).

The rate of blinking was found to be negatively correlated to the TMH, again with no statistical significance (*P* = 0.14).

A summary of the correlation between the different studied parameters in Group B is elaborated in Table 3.

## 4. Discussion

Diabetes mellitus is a systemic disease that affects mainly the microcirculation and can affect the ocular surface integrity through different mechanisms [11].

The prevalence of dry eye in diabetic patients has been reported to be about 50% in type 2 diabetes. 7% of children with type 1 diabetes were reported to have dry eye manifestations compared to 0% of age matched control children [2].

In the present study, we tried to study the impact of diabetes on the tear film quality and quantity in diabetic patients with no subjective symptoms of dry eye.

We also tried to find a correlation between the different studied tear film parameters in diabetics versus normal controls.

In the present study, the mean TTT was found to be less in the diabetic group as compared to the control group, although not reaching a statistically significant value; this finding agrees with multiple previous studies, which concluded that diabetes can affect the tear film stability [9, 12, 13].

The reason why we preferred to perform the TTT over the regular tear break-up test (TBUT) with fluorescein is the postulated limitations, including the need to instill fluorescein dye, lack of standardization of fluorescein concentration or amount, and the possible induction of reflex tearing that might alter the results of the test [5].

TMH is typically used to detect the amount of the aqueous layer of the tear film. In our study it was noted to have a mean value higher in the diabetic than in the control group, yet with no statistical significance. Again, this agreed with previous published results that diabetic patients might show a decrease in BUT, a decrease in basal secretion yet with a normal overall tear secretion [9].

Diabetes is one of the well-established causes of excessive blinking (blinking eye syndrome) [11]. However, some authors reported a decrease in the blinking rate in diabetics but with an increase in the interblinking interval [14].

In our study, the rate of blinking per minute was found to be significantly higher in the diabetic as compared to the control group. We owe this to the changes in ocular surface integrity (namely, recurrent epithelial defects) associated with diabetes with subsequent irritation. Furthermore, blinking was found to be significantly higher in females as compared to males within the diabetic group. This gender difference in blinking rate needs to be further investigated in more detail, as other hormonal factors may be involved.

Several studies demonstrated that dry eye could be correlated with the duration of diabetes as well as the severity of retinopathy [15, 16]; however, others postulated that the metabolic control might be of great impact rather than the duration [2, 8].

In our study, we did not study the correlation with the severity of the disease; however, a statistically significant impact could be elicited regarding the effect of duration of diabetes on tear production (i.e., TMH) as well as on the rate of blinking and the TTT. All values showed further decrease with increasing duration of diabetes.

The absence of subjective symptoms of dryness (as all our patients did not complain of dry eye symptoms) despite the low values of TTT in the diabetic group can be explained by the fact that the associated decrease in corneal sensitivity caused by diabetic peripheral neuropathy in those patients might mask the symptoms of dryness.

Previous studies demonstrated that the impact of diabetes on ocular surface integrity and subsequently on dry eye associated with the disease might be affected with the decreased circulating sex hormones in the postmenopausal diabetic females, a thing that might add to the severity of the condition [11]. This point was not investigated in depth in our work. However, we noted that the TTT values were found to be less in diabetic females as compared to males, despite higher TMH values. Whether this is or is not related to menopause or is merely a gender difference needs to be further investigated in detail in subsequent studies.

The mechanisms by which diabetes affects the integrity of the ocular surface, and the tear film stability and tear production have been previously investigated, whereas the impact of diabetes might also be related to the microcirculation of the lacrimal gland, a point which needs to be further correlated with tear film parameters [17, 18].

Some studies suggested that the decreased corneal sensitivity and corneal neuropathy to be a cause of decreased basal tear secretions in diabetics [16]. Moreover, an inflammatory theory, with the postulation that dry eyes might be secondary to an inflammatory process mediated through T-call lymphocytes was also suggested by some authors [9].

In conclusion, our study showed that the tear film integrity was found to be affected in diabetic patients, as compared to age matched controls, especially when it comes to the blinking rate. A significant negative correlation was found between the duration of diabetes and all studied tear film parameters. The correlation between the volume and stability of the tear film in diabetics was found to be a positive one. A significant difference in blinking rate was found between males and females in the diabetic group, the exact cause of which needs further investigation.

The associated neuropathy with the secondary decrease in corneal sensitivity might add to the severity of the condition, yet with minimal symptoms. Our study thus recommends that a routine examination for dry eye should be considered in all diabetic patients even in the absence of subjective symptoms.

In diabetics the tear film stability might be affected, despite an increase in tear production that is, TMH, due to the fact that diabetes can affect the basal tear production mainly, rather than the total tear production. This again should not trick us into excluding dry eye in a diabetic patient depending only on the normal TMH measurements [9].

The mechanism of dry eye in diabetes is a multifactorial one, where the diagnosis should be based on the measurement of multiple tear parameters rather than a single one, as not all the parameters are affected in a similar pattern.

The severity of the condition might be related to the severity of diabetes and the metabolic control of the disease; however, the duration of the disease plays a significant role as well.

## Figures and Tables

**Table 1:** Demographic data of patients.

	Group A	Group B	*P* value
Age	40–70 yrs (57.27 ± 8.5)	30–77 yrs (57.12 ± 12.22)	0.871

Females	13 (59.1%)	9 (56.2%)	0.861
Males	9 (40.9%)	7 (43.8%)	

**Table 2:** The cross relations in Group A.

		Age	TTT	Blinks/min	TMH	DM duration
Age	Correlation coefficient		−0.096	−0.396	−0.625	0.470
	*P*		0.670	**0.068**	*0.002*	*0.027*

TTT	Correlation coefficient	−0.096		0.047	0.238	−0.208
	*P*	0.670		0.836	0.286	0.353

Blinks/min	Correlation coefficient	−0.396	0.047		0.239	−0.446
	*P*	**0.068**	0.836		0.285	*0.037*

TMH	Correlation coefficient	−0.625	0.238	0.239		−0.462
	*P*	*0.002*	0.286	0.285		*0.030*

DM duration	Correlation coefficient	0.470	−0.208	−0.446	−0.462	
	*P*	*0.027*	0.353	*0.037*	*0.030*	

**Table 3:** The cross relations in Group B.

		Age	TTT	Blinks/min	TMH
Age	Correlation		−0.155	0.025	−0.028
	*P*		0.567	0.928	0.917

TTT	Correlation	−0.155		−0.218	0.466
	*P*	0.567		0.418	0.069

Blinks/min	Correlation	0.025	−0.218		−0.383
	*P*	0.928	0.418		0.143

TMH	Correlation	−0.028	0.466	−0.383	
	*P*	0.917	**0.069**	0.143	
